# From Clinical Clues to Final Diagnosis: The Return of Detective Work to Clinical Medicine in Cardiac Amyloidosis

**DOI:** 10.3389/fcvm.2021.644508

**Published:** 2021-06-25

**Authors:** Hani Sabbour, Khwaja Yousuf Hasan, Firas Al Badarin, Haluk Alibazoglu, Andrew L. Rivard, Ingy Romany, Stefano Perlini

**Affiliations:** ^1^Cleveland Clinic Abu Dhabi, Abu Dhabi, United Arab Emirates; ^2^Warren Alpert School of Medicine, Brown University, Providence, RI, United States; ^3^Pfizer Gulf FZ LLC, Dubai, United Arab Emirates; ^4^Emergency Department, Amyloid Research and Treatment Center, Istituto di Ricovero e Cura a Carattere Scientifico (IRCCS), Policlinico San Matteo Foundation, Internal Medicine Department, University of Pavia, Pavia, Italy

**Keywords:** amyloidosis, ATTR, transthyretin amyloid cardiomyopathy, immunoglobulin light chain amyloidosis, differential diagnosis

## Abstract

Cardiac amyloidosis is frequently misdiagnosed, denying patients the opportunity for timely and appropriate management of the disease. The purpose of this review and case studies is to raise awareness of the diagnostic “red flags” associated with cardiac amyloidosis and the currently available non-invasive strategies for diagnosis. The review focuses on the identification of one of the two main types of cardiac amyloidosis, transthyretin amyloid cardiomyopathy, and non-invasive tools to distinguish this from light-chain amyloidosis. A diagnostic algorithm centered around the use of non-invasive imaging and laboratory analysis is presented. The algorithm generates four differential diagnoses for patients presenting with signs and symptoms consistent with cardiac amyloidosis. Case examples are presented, representing the four potential outcomes of diagnosis using the algorithm. The review provides a guide on how to recognize the often-overlooked presentations of this disease in clinical practice. Non-invasive imaging techniques and diagnostic tools that do not require the involvement of a specialty center have allowed for the improved diagnosis of cardiac amyloidosis. Timely diagnosis of this life-threatening disease is essential for optimal management and it is imperative that clinicians have a high index of suspicion for patients presenting with “red flag” symptoms.

## Introduction

Amyloidosis is a multisystem, progressive and life-threating disorder caused by the deposition of misfolded fibrillar proteins that form insoluble amyloid fibrils in the extracellular matrix of various organs including the heart (cardiac amyloidosis), peripheral and autonomic nervous system, soft tissues as well as kidneys and gastrointestinal (GI) tract (1–4).

Amyloidosis presents a clinical challenge for clinicians, from initial suspicion to identifying the type of amyloidosis due to the complex multisystem manifestations of the disease. Patients may initially present to clinicians in multiple specialties who are highly focused in siloed disciplines (5). The diagnosis is equally challenging since it requires diagnostic tests more familiar to hematologists coupled with a lack of awareness that cardiac biopsy is no longer necessary for diagnosis. The variability in initial patient presentations coupled with a lack of integration of subspecialties and a complex diagnostic algorithm makes it mandatory for physicians to raise the suspicion of diagnosis by searching for clues for amyloidosis (5).

There is limited epidemiologic data on the incidence and prevalence of systemic amyloidosis. A 2013 study from the UK estimates the incidence to exceed 8.0 per million inhabitants per year (6). In a population-based study in Finland, the prevalence of senile systemic amyloidosis is reported to be as high as 25% in the elderly population (7). The prevalence of light chain (AL) amyloidosis in the United States increased significantly between 2007 and 2015, from 15.5 cases per million in 2007 to 40.5 cases in 2015 (8). More recently, the incidence of AL amyloidosis from Italy in 2021 is estimated at 9 cases per million person-years (9). From recent reports, ATTR wild type cardiac amyloidosis appears to be quite common. Contemporary diagnostic strategies indicate that the prevalence ranges from 13% among older patients having heart failure with preserved ejection fraction (HFpEF) to 16% in patients with aortic stenosis undergoing transcatheter aortic valve replacement (10, 11).

The two most common types of cardiac amyloidosis are caused by deposition of misfolded transthyretin protein, resulting in transthyretin amyloid cardiomyopathy (ATTR-CM), or by deposition of abnormal circulating immunoglobulin light chains, resulting in AL amyloidosis (2, 3, 12, 13). Both ATTR-CM and AL amyloidosis result in a progressive infiltrative/restrictive cardiomyopathy, predominantly presenting as heart failure, arrhythmia, atrial fibrillation, and atrioventricular (AV) block. However, their management differs considerably, with AL considered a hematological emergency due to its aggressive nature and poor survival measured in weeks to months (2, 3, 13–16). Differentiating between ATTR-CM and AL is vital for the delivery of appropriate treatment.

### Subtypes of ATTR-CM

There are two subtypes of ATTR-CM and these are classified by the sequence of the transthyretin (*TTR*) gene; wild-type, with no mutation (ATTRwt), or hereditary, with a single-point mutation (ATTRm) (15). Both subtypes may result in the deposition of the same abnormal amyloid protein within the myocardium; however, organ involvement and age of onset vary considerably between the two subtypes (15).

#### Wild-Type Transthyretin Amyloidosis (ATTRwt)

ATTRwt amyloidosis, formerly called senile systemic amyloidosis, results from age-related changes in wild-type *TTR* stability (3). ATTRwt is thought to be the most common cause of cardiac amyloidosis, particularly in the elderly, affecting up to 10% of elderly patients with heart failure (3). While the exact prevalence of ATTRwt is unknown, autopsy studies suggest that ~25% of individuals over the age of 80 years have wild-type TTR fibrils in their myocardium, regardless of whether or not they exhibited symptoms of the disease (7). The median reported survival following a diagnosis of ATTRwt ranges from 43 to 67 months (3). Therefore timely diagnosis is of the utmost importance.

While the heart is the most common organ involved in ATTRwt amyloidosis, deposition of amyloid fibrils also occurs in ligaments and tendons, resulting in unusual symptoms of bilateral carpal tunnel syndrome, ruptured biceps tendons, and lumbar spinal stenosis (1, 12). These symptoms often precede cardiac symptoms by several years and should be an important clue in the diagnosis of cardiac patients (12).

#### Hereditary (Mutant) Transthyretin Amyloidosis (ATTRm)

ATTRm amyloidosis is an autosomal dominant condition with variable penetrance that commonly involves the nervous system as well as the heart (12). The phenotypic penetrance of ATTRm varies significantly with genetic mutation, age at the time of onset, and geographical location (12, 13). ATTRm has a more aggressive presentation and the median reported survival from diagnosis ranges from 26 to 62 months (3).

While there are over 120 identified pathogenic mutations of the *TTR* gene in ATTRm, three particular *TTR* variants, Val122Ile, Leu111Met, and Ile68Leu, predominantly affect the heart (12, 13). The most common variant in the US is Val122Ile, which occurs in ~3–4% of African Americans (13). This variant leads to ATTR with a clinical onset approximately a decade earlier than that seen with ATTRwt and involves a higher proportion of female patients (12).

### Risk of Delayed Recognition of ATTR-CM

ATTR-CM is associated with poor life expectancy, usually 2–6 years post diagnosis (13). The prognosis of ATTR-CM worsens rapidly with the continued deposition of amyloid and treatment is most effective when administered before significant symptoms (New York Heart Association Class III–V) or dysfunction manifest (1, 3).

Notably, failure to consider cardiac amyloidosis is an important reason for delaying treatment. Cardiac amyloidosis is often misdiagnosed for non-amyloid HFpEF, hypertensive heart failure, or hypertrophic cardiomyopathy (1, 13, 17). A 2017 survey of ATTR-CM patients revealed a misdiagnosis in over 39% of patients, with 17% visiting five different physicians before receiving the correct diagnosis; 75% received treatment for their misdiagnosis (18). Due to the restrictive physiology, routine use of heart failure guideline-directed medical therapy (including beta blockers, angiotensin-converting enzyme inhibitors, angiotensin II receptor antagonists and angiotensin receptor/neprilysin inhibitors [ARNI]) are poorly tolerated by patients with cardiac amyloidosis, this intolerance presents a clue to the diagnosis (19). Delays in diagnosing cardiac amyloidosis could be associated with worsening symptoms and quality of life for patients (3).

Recent advances in readily accessible imaging techniques, including nuclear imaging with radiotracers used for bone scintigraphy, that allow for non-invasive diagnosis outside of specialist centers, have improved the identification of ATTR-CM (3, 13, 20, 21). Clear frameworks for the diagnosis and management of ATTR-CM are now available and a number of “red flags” have been identified that can raise suspicion for the presence of this disease (3, 21). This review and presentation of case studies aims to raise awareness of diagnostic “red flags” associated with cardiac amyloidosis and the currently available non-invasive strategies for diagnosing this condition, while emphasizing the importance of maintaining a high index of suspicion in patients presenting with relevant symptoms. An algorithm to simplify the evaluation of patients with suspected diagnosis of cardiac amyloidosis is also presented.

## Clues in Identifying ATTR-CM

ATTR-CM is typically a late-onset disease with symptoms predominantly manifesting in males over the age of 65 years (3). The typical presentation of ATTR-CM includes symptoms of heart failure (dyspnea on exertion, fatigue, orthostatic hypotension, syncope and edema) or arrhythmias due to involvement of the cardiac conduction system (1, 3, 22). Unusual extracardiac signs that must be actively elicited or identified in the patients' history include neurological, ophthalmological and GI symptoms, or a history of carpal tunnel syndrome or biceps tendon rupture (1, 3, 22). As the clinical findings are often non-specific, a high index of suspicion is the key to diagnosis (1).

**Figure 1:**
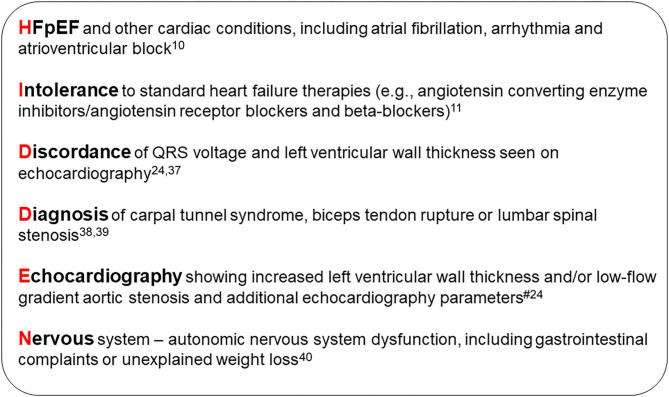
HIDDEN mnemonic. ^#^Echocardiographic, speckle-strain and tissue doppler “red flags”: Heart failure normal or mid-range ejection fraction; increased wall thickness; left atrial enlargement; low stroke volume index; low-flow, low-gradient aortic stenosis (Stage D2); low myocardial contraction fraction; advanced diastolic dysfunction; impaired global longitudinal strain with apical sparing; low mitral annular tissue Doppler S' (average septal and lateral annulus) (24). HFpEF, heart failure with preserved ejection fraction.

An international group of experts have devised a clear definition of which patients to target for screening for ATTR-CM (3). Fundamental to this is the use of echocardiography for the assessment of myocardial wall thickness; individuals with a wall thickness ≥12 mm and evidence of heart failure or “red flag” symptoms should be considered for evaluation based on gender and age (3, 15, 23). The “HIDDEN” mnemonic is a useful tool to help remember some of the commonly encountered “red flags” associated with ATTR-CM (Figure 1).

Other potential indicators of ATTR-CM not included in the “HIDDEN” mnemonic that are also useful in the right clinical context include: (1, 3, 25).

- Intolerance to antihypertensive medications because of symptomatic hypotension or orthostasis
- Spontaneous resolution of hypertension
- Marked extracellular volume expansion, abnormal nulling time for the myocardium or diffuse late gadolinium enhancement on cardiac magnetic resonance imaging (MRI)
- AV block, in the presence of increased left ventricular wall thickness
- Persistent low-level elevation in serum troponin
- Previous pacemaker implantation
- Family history of cardiomyopathy
- Erectile dysfunction/impotence
- Gastroparesis
- Postprandial diarrhea alternating with constipation
- Orthostatic hypotension
- Urinary retention/incontinence
- Lumbar spinal stenosis
- Unprovoked biceps tendon rupture
- Hip and knee arthroplasty
- Family history or polyneuropathy.

### Non-invasive Definitive Diagnosis of ATTR-CM and Exclusion of AL Amyloidosis

**Figure 2:**
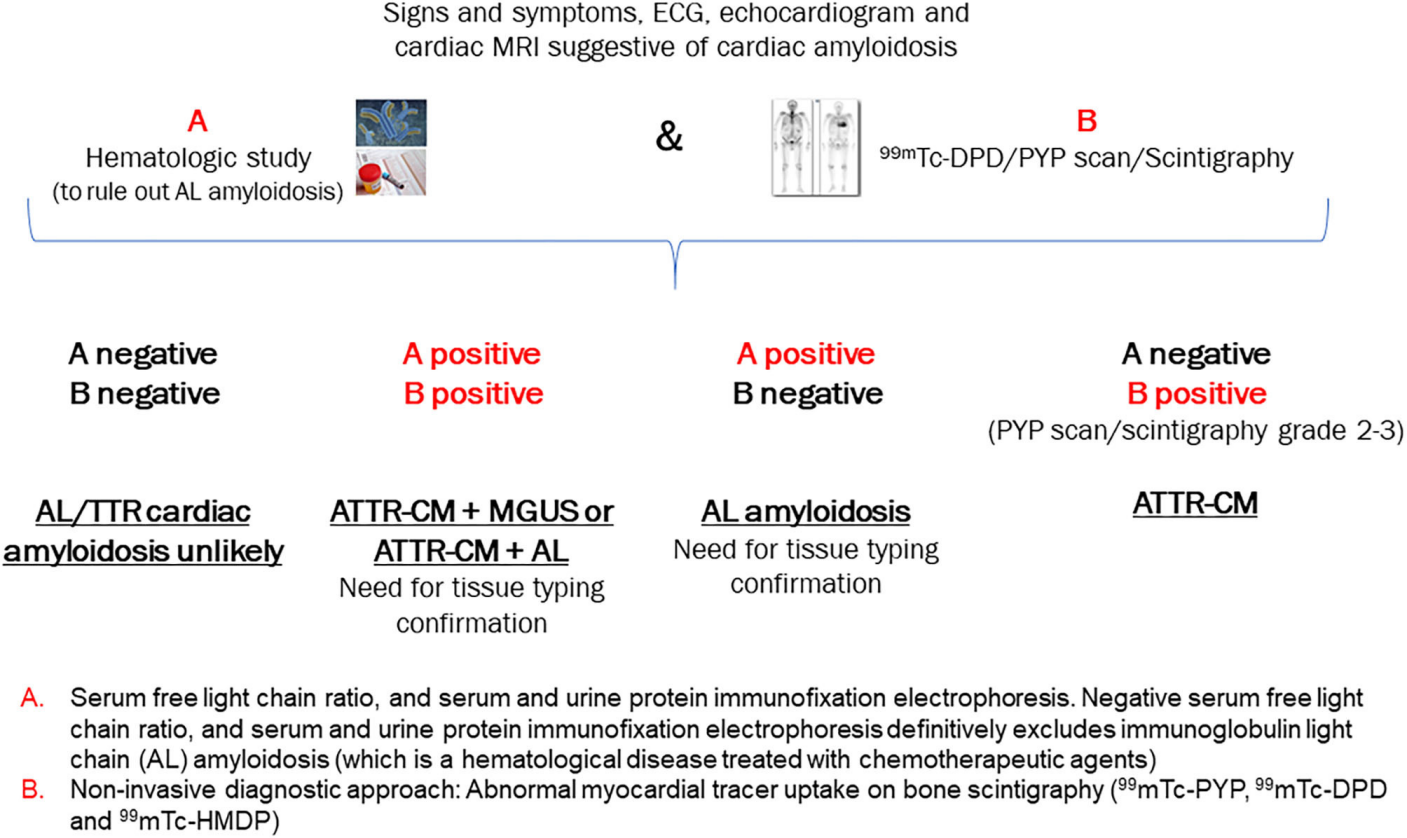
Algorithm for the non-invasive diagnosis of ATTR-CM. [Adapted from Gillmore 2016] (21). AL, light-chain amyloidosis; ATTR, transthyretin amyloidosis; ECG, electrocardiogram; MRI, magnetic resonance imaging; PYP, pyrophosphate; ^99m^TC-DPD, technetium-99m-3, 3-diphosphono-1, 2 propanodicarboxylic acid.

If a patient presents with several of the “red flags” for ATTR-CM and a high clinical index of suspicion has been established, timely diagnostic assessment of ATTR-CM is essential (3, 23). A historical barrier to early specific diagnosis was the need for endomyocardial biopsy; however, the current algorithm described below (Figure 2) obviates this need in the majority of patients (26). Nuclear scintigraphy is the cornerstone of ATTR-CM diagnosis; however, this modality alone cannot distinguish between ATTR-CM and AL amyloid cardiomyopathy (1). In most cases, by combining the results of serum/urine tests to rule out AL amyloidosis and the findings of nuclear scintigraphy, it is possible to achieve a non-biopsy, non-invasive diagnosis of ATTR-CM, as detailed in the algorithm in Figure 2 (3, 13, 21).

### Serum/Urine Tests to Rule Out AL Amyloidosis

The first priority is to rule out AL amyloidosis, since it is considered a life-threatening hematological emergency (3, 27). Patients with AL amyloidosis have poor survival (<6 months) if untreated, but there are a number of chemotherapeutic and disease-modifying therapies available for the management of this condition (28, 29).

AL amyloidosis is indicated by the presence of a monoclonal protein in the serum or urine, that is amyloidogenic (3). Monoclonal protein is also present in patients with monoclonal gammopathy of undetermined significance (MGUS), a premalignant disorder (30). It is estimated that up to 8% of the population over 65 years exhibit MGUS and up to 40% of patients with ATTR-CM may also have this condition (3, 13).

The following tests should be ordered to screen for monoclonal protein (13):

- Serum kappa/lambda free light chain ratio (abnormal if ratio is <0.26 or >1.65^*^)
- Serum immunofixation electrophoresis (abnormal if monoclonal protein is detected)
- Urine immunofixation electrophoresis (abnormal if monoclonal protein is detected)

Mild elevations in the serum kappa/lambda free light chain ratio are common in patients with kidney disease and when these patients exhibit normal serum and urine immunofixation electrophoresis, a kappa/lambda ratio up to 2.5 can be considered normal (31).

Together these three tests give >99% sensitivity for the detection of AL amyloidosis (1). It should be noted that conventional serum/urine protein electrophoresis is insensitive and not recommended for this application.

If monoclonal protein is detected (suggesting AL amyloidosis or MGUS), the patient should rapidly be referred for a comprehensive hematological evaluation, including tissue biopsy from a clinically affected organ, bone marrow or abdominal fat, to confirm the diagnosis and to further typify amyloid protein. In ATTR, fat pad biopsy carries a high false-negative rate, or low sensitivity, and further evaluation is warranted in case of negative biopsy with high clinical suspicion (13). Endomyocardial biopsy with Congo red staining, immune-electron microscopy or mass-spectrometry proteomics is the gold standard in cases of equivocal non-invasive findings, with tissue typing to identify the nature of the deposited amyloid fibril (13, 32).

If monoclonal protein is not detected (normal serum kappa/lambda free light chain ratio or no monoclonal protein in serum/urine) then AL amyloidosis is very unlikely (14).

### Nuclear Scintigraphy

Once AL amyloidosis has been ruled out, the next step in non-invasive diagnosis of ATTR-CM is nuclear scintigraphy using bone-avid radiotracers (15). This technique has revolutionized the diagnosis of ATTR-CM. While the exact mechanism by which the radiotracer localizes to TTR cardiac amyloid is not fully understood, this imaging modality has proven to be an affordable, non-invasive and highly sensitive tool in the diagnosis of ATTR-CM (3, 14). Studies using ^99m^technetium-labeled pyrophosphate (^99m^Tc-PYP) have demonstrated that cardiac TTR can be identified using semiquantitative assessment of heart to contralateral lung ratio (H/CL) with a ratio of ≥1.5, giving a sensitivity of 97% and specificity of 100% (33). From a clinical perspective, confusion may arise when there is uptake in the myocardium of patients with AL amyloid. However, in the absence of monoclonal protein on serum/urine testing, bone scintigraphy has a specificity of 100% for detecting ATTR-CM when Grade 2 or Grade 3 radiotracer uptake is seen (3).

Planar imaging is usually undertaken initially, followed by single-photon emission computed tomography (SPECT) imaging if there is evidence of myocardial uptake (33). There are three commonly used and validated radiotracers: ^99m^Tc-PYP, ^99m^technetium-labeled 3,3-diphosphono-1,2-propanodicarboxylic acid (^99m^Tc-DPD) and ^99m^technetium-labeled hydroxymethylene diphosphonate (^99m^Tc-HMDP) (3, 14).

Cardiac uptake of radiotracer in the assessment of cardiac TTR may be classified according to comparison with tracer uptake in the rib bone: (1, 21).

- Grade 0 = no myocardial uptake with rib uptake
- Grade 1 = myocardial uptake less than rib uptake
- Grade 2 = myocardial uptake equal to rib uptake
- Grade 3 = myocardial uptake higher than rib uptake

It should be noted that in addition to Grade 0 and Grade 1 uptake which may be observed in AL amyloidosis, Grade 2 or Grade 3 uptake can also be seen in >20% of patients with AL cardiac amyloidosis (34). However, AL amyloidosis would have been excluded through negative serum/urine light-chain analysis.

Since the diagnostic pathway is relatively complex with multiple decision points, an attempt has been made to simplify this for the clinician (Figure 2) (35). The application of this algorithm to cases with confirmed clinical diagnosis will be used to highlight how to best utilize the pathway in clinical practice.

## Clinical Algorithm Integrating Diagnostic Tools

Using the clinical algorithm (Figure 2), **If A is negative and B is negative**: Both AL cardiac amyloidosis and ATTR-CM are ruled out. However, if suspicion of cardiac amyloidosis remains high based on other clinical findings, biopsy of an affected organ is recommended.

**If A is positive and B is positive:** Overlapping cases in which tissue biopsy is required to exclude AL amyloidosis definitively

- ATTR-CM + MGUS (bone marrow biopsy required)—MGUS should be followed up as it may convert to multiple myeloma (Case 1).
- ATTR-CM + AL amyloidosis (very rare; bone marrow biopsy and possibly endomyocardial biopsy to determine which of the two [if not both] is depositing proteins in the heart; if other organs are affected such as skin or kidneys, then these may be biopsied and offer a safer option than cardiac biopsy).

**If A is positive and B is negative:** AL amyloidosis

- If any of the hematological tests (serum free light chain ratio, serum and urine protein immunofixation electrophoresis) are positive and the bone scintigraphy scan is negative, AL amyloidosis is suspected and referral for bone marrow biopsy is essential. Chemotherapy is to be initiated if the biopsy is positive (Case 2).

**If A is negative and B is positive:** ATTR-CM

- When grade 2–3 myocardial radiotracer uptake of ^99m^Tc-PYP, ^99m^Tc-DPD or ^99m^Tc-HMDP is evident and the hematological tests are negative, there is an almost 100% likelihood of ATTR-CM, negating the need for cardiac biopsy (Cases 3 and 4). Genetic sequencing of the *TTR* gene is required to determine hereditary vs. wild-type disease and to inform genetic counseling and screening of family members if hereditary disease is present.
- In the event that PYP presence is equivocal, the patient should be followed up for further assessment and the scan should be repeated within 6 months as this may represent early disease (36).

### Case Examples

Throughout the case descriptions, “red flag” symptoms and signs suggestive of cardiac amyloidosis are highlighted in bold. The presence of commonly encountered “red flag” symptoms associated with ATTR-CM for each of the cases are summarized in Table 1.

**Table 1:** Summary of commonly encountered “red flag” symptoms associated with ATTR-CM from the HIDDEN mnemonic present in each illustrative case.

**“Red flag” symptoms associated with ATTR-CM**	**Case 1 ATTR-CM + MGUS**	**Case 2 AL amyloidosis**	**Case 3 ATTRwt-CM**	**Case 4 ATTRm-CM**
**HFpEF** and other cardiac conditions, including atrial fibrillation, arrhythmia and atrioventricular block (10)	**✓**	**✓**	**✓**	
**Intolerance** to standard heart failure therapies (e.g., angiotensin converting enzyme inhibitors/angiotensin receptor blockers and beta-blockers) (19)		**✓**	**✓**	
**Discordance** of QRS voltage and left ventricular wall thickness seen on echocardiography (24, 37)	**✓**	**✓**	**✓**	**✓**
**Diagnosis** of carpal tunnel syndrome, biceps tendon rupture or lumbar spinal stenosis (38, 39)	**✓**	**✓**	**✓**	**✓**
**Echocardiography** showing increased left ventricular wall thickness and/or low-flow gradient aortic stenosis and additional echocardiography parameters^#^ (24)	**✓**	**✓**	**✓**	**✓**
**Nervous system**—autonomic nervous system dysfunction, including gastrointestinal complaints or unexplained weight loss (40)		**✓**	**✓**	**✓**

# *Echocardiographic, speckle-strain and tissue doppler “red flags”: Heart failure normal or mid-range ejection fraction; increased wall thickness; left atrial enlargement; low stroke volume index; low-flow, low-gradient aortic stenosis (Stage D2); low myocardial contraction fraction; advanced diastolic dysfunction; impaired global longitudinal strain with apical sparing; low mitral annular tissue Doppler S' (average septal and lateral annulus) (24)*.

*AL, light-chain amyloidosis; ATTR-CM, transthyretin amyloid cardiomyopathy; ATTRm-CM, hereditary (mutant) transthyretin amyloid cardiomyopathy; ATTRwt-CM, wild-type transthyretin amyloidosis cardiomyopathy; HFpEF, heart failure with preserved ejection fraction; MGUS, monoclonal gammopathy of undetermined significance; QRS, Q wave, R wave, and S wave*.

#### Case 1: ATTR-CM +MGUS (A Is Positive and B Is Positive)

##### Presentation

A **77-year-old man** was evaluated for severe tricuspid and mitral regurgitation along with eosinophilia. He reported a several-month history of worsening fatigue and shortness of breath on exertion, increasing abdominal swelling, and progressively increasing bilateral pedal edema. He had **right-sided heart failure**, **spinal stenosis**, **peripheral neuropathy** (upper and lower limbs), a chronic cough, hoarseness of voice, and his mobility had become significantly restricted. He denied symptoms of chest pain or syncope. His past medical history was significant for essential hypertension, hyperlipidemia, chronic kidney disease and coronary artery disease with a history of percutaneous coronary intervention.

##### Investigations

**Figure 3:**
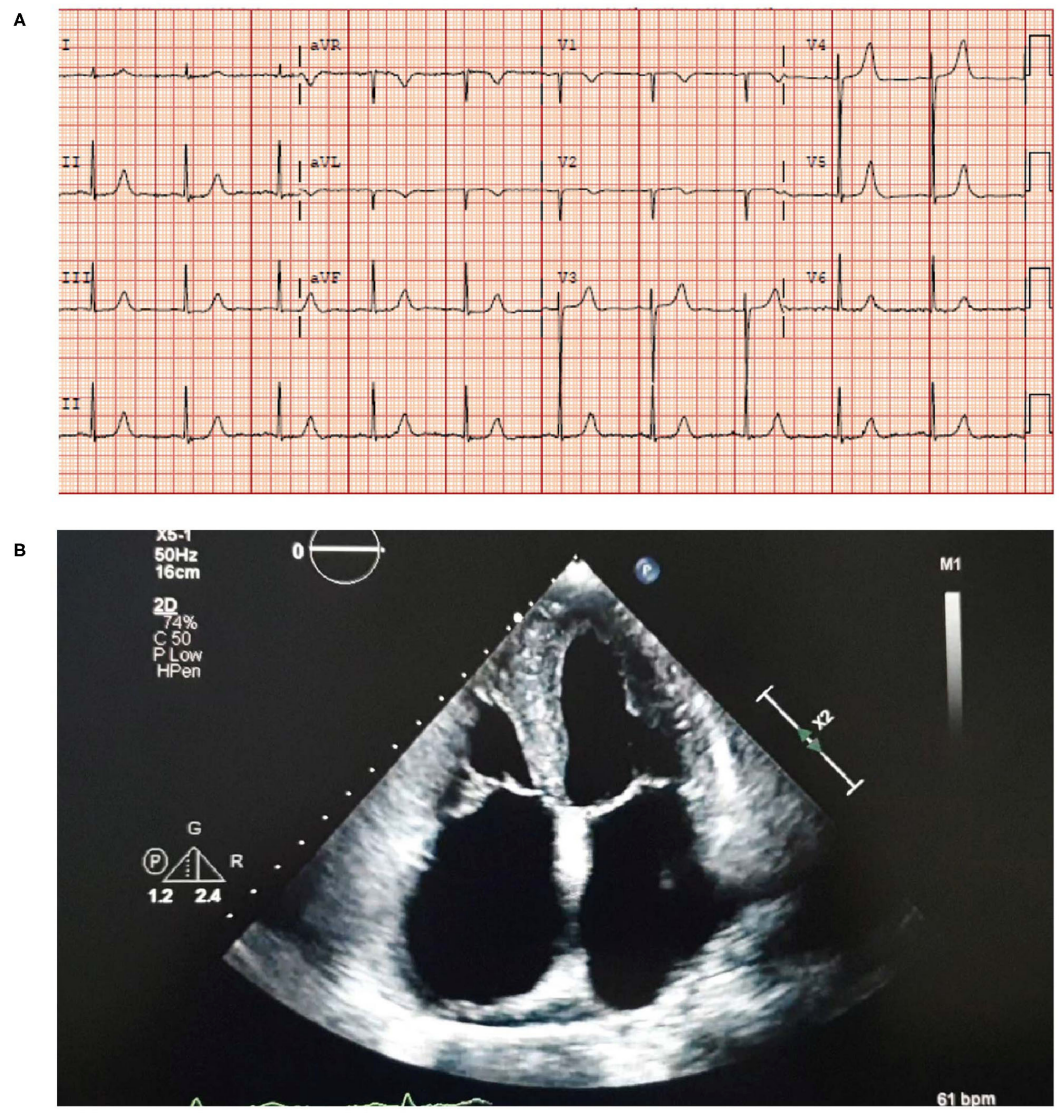
ECG **(A)** showing sinus rhythm with LVH and a borderline prolonged QT interval. Echocardiogram **(B)** showing moderate concentric LVH, moderate to severe biatrial enlargement and mild RV enlargement. ECG, electrocardiogram; LVH, left ventricular hypertrophy; RV, right ventricular.

**Figure 4:**
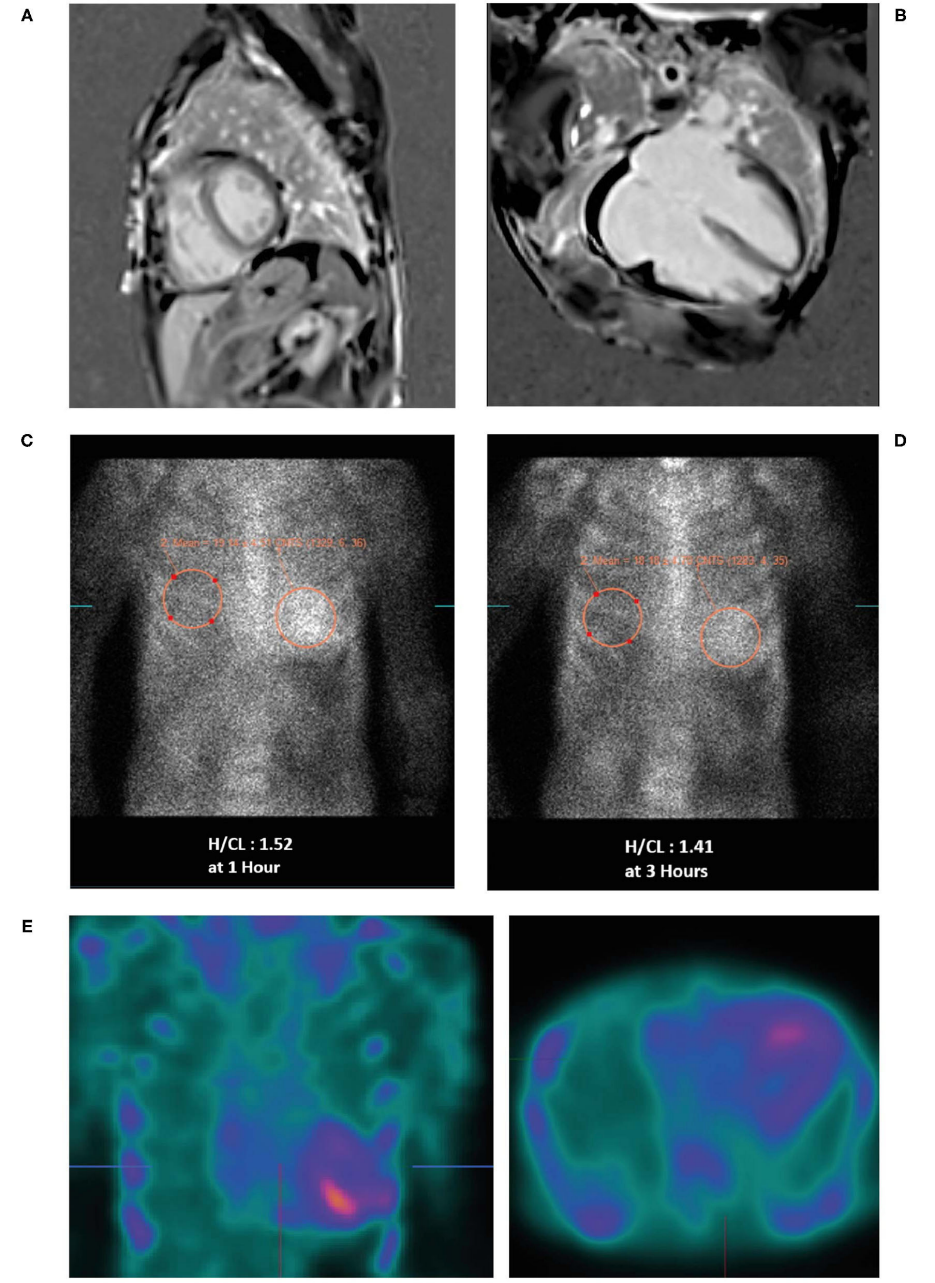
Cardiac MRI **(A,B)** showing biatrial enlargement without evidence of infiltrative disease. Planar bone scintigraphy (^99m^Tc-PYP) at 1 h **(C)** and 3 h **(D)** showing myocardial PYP uptake greater than rib uptake (grade 3) via semi-quantitative assessment—strongly suggestive of ATTR-CM. SPECT scan **(E)** at 1 h showing no blood pool. ^99m^Tc-PYP, ^99m^technetium-labeled pyrophosphate; ATTR-CM, transthyretin amyloid cardiomyopathy; MRI, magnetic resonance imaging; PYP, pyrophosphate; SPECT, single-photon emission computed tomography.

- Physical examination revealed bilateral pedal edema up to the knees, a pan systolic murmur at the left lower sternal border and shifting dullness consistent with ascites.
- **An electrocardiogram (ECG) showed sinus rhythm with left ventricular hypertrophy (LVH) and a borderline prolonged QT interval** (Figure 3A).
- Laboratory results were: Creatinine 156 μmol/L, eGFR 36 ml/min/1.73 m^2^, hemoglobin 113 g/L and normal red cell indices. Total leucocyte count was 4,040 cells/μL with a low absolute neutrophil count of 1,400 cells/μL and an elevated absolute eosinophil count of 1,000 cells/μL. **NT proBNP was 3,843 ng/L and troponin levels were 0.046** **μg/L**.
- An echocardiogram revealed moderate concentric **LVH** with an ejection fraction (EF) of 52%, **moderate to severe biatrial enlargement and mild right ventricular (RV) enlargement** (Figure 3B). Moderately severe mitral valve regurgitation with an effective regurgitant orifice area proximal isovelocity aarea (EROA [PISA]) of 0.32 cm^2^ and severe tricuspid regurgitation caused by annular dilation and complete non-coaptation of the tricuspid valve leaflets with systolic flow reversal in the hepatic veins. The inferior vena cava was dilated with minimal respiratory collapse.
- Of note, the absence of ECG and echocardiogram discordance does not exclude ATTRwt and further investigations are deemed necessary (Figure 3) (41).
- A cardiac MRI was undertaken (Figures 4A,B) due to suspicion of infiltrative cardiomyopathies such as amyloidosis and sarcoidosis. The MRI did not show any evidence of scar or infiltrative disease. Nevertheless, due to the strong clinical suspicion, a ^99m^Tc-PYP scan was ordered. Of note, a negative cardiac MRI does not exclude a diagnosis of cardiac amyloidosis.
- ^**99*m***^**Tc-PYP scans revealed an H/CL ratio of 1.52 at 1 h and 1.41 at 3 h** (Figures 4C,D), highly suggestive of ATTR-CM. No blood pool was seen on 1-h SPECT imaging (Figure 4E).
- Simultaneous urine and serum immunofixation studies and free light chain analysis were performed. Immunofixation showed IgA levels of 395 mg/dL, IgG levels of 1,811 mg/dL (slightly elevated) and IgM levels of 85 mg/dL. Beta-2 microglobulin was high at 4.91 mg/dL. Urine immunofixation was normal. Serum free kappa light chain level was 152.26 mg/L, free lambda light chain levels were 36.54 mg/L, and the serum kappa/lambda ratio was 4.17. Marked elevation of the free light chain kappa/lambda ratio was noted.
- Bone marrow biopsy revealed mild plasmacytosis (<3%) and negative amyloid staining. The findings were consistent with MGUS.
- A fat pad biopsy was negative for Congo red staining.

##### Diagnosis

These findings were highly suggestive of ATTR-CM associated with MGUS.

##### Clinical Clues and Potential Pitfalls

MGUS is common in the elderly population, with a prevalence of 5.3% in persons aged ≥70 years (42). In this situation, an invasive biopsy to establish tissue diagnosis is mandatory.

#### Case 2: AL Amyloidosis (A Is Positive and B Is Negative)

##### Presentation

**Figure 5:**
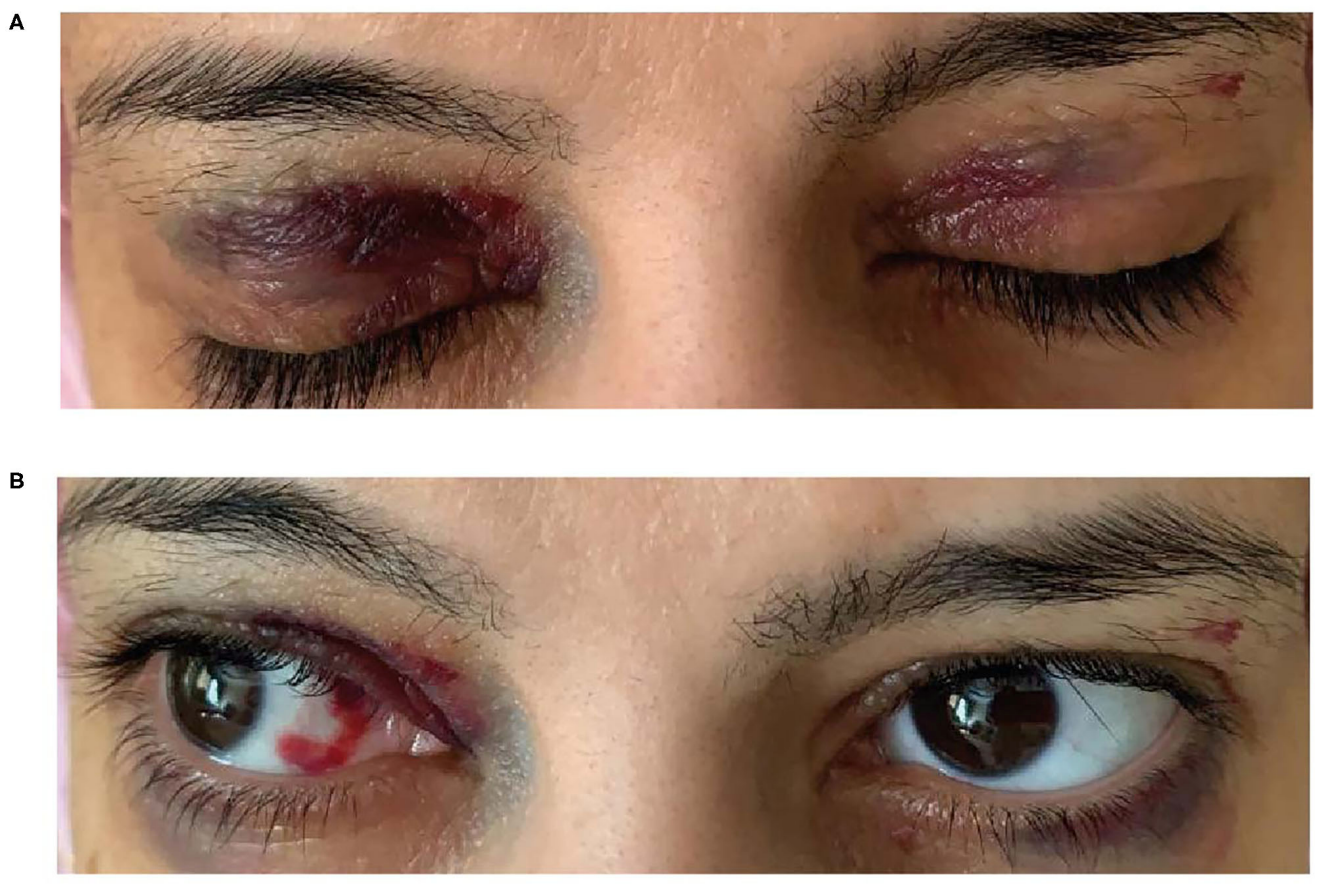
Bilateral heliotrope rash **(A)** Spontaneous subconjunctival ecchymosis **(B)**.

A 38-year-old woman with a **history of back pain** and **bilateral carpal tunnel syndrome** presented to the emergency department after a vasovagal spell. She reported experiencing **chronic fatigue** during the previous few months but denied a history of chest pain or **shortness of breath**. She experienced epigastric pain, **constipation, weight loss** and was **anemic**; iron studies were normal as were B12 and vitamin D. She experienced dizziness, macroglossia, a hoarse voice and bleeding spots around her eyes (heliotrope rash) (Figures 5A,B)—a “red flag” for AL amyloidosis, hence AL amyloidosis diagnosis was suggested. Her dizziness subsequently worsened, and she experienced abdominal pain, **syncope**, hypotension with worsening anemia and more frequent **periorbital ecchymosis and bilateral subconjunctival hemorrhage**. The patient was unable to tolerate the lowest dose of angiotensin-converting-enzyme inhibitors due to orthostasis and hypotension with syncope, indicating an **intolerance to guideline-directed medication for heart failure**. Subsequent developments included **loss of consciousness** with tingling in her left arm but no evidence of cerebrovascular accident. She had **edema and fluid overload** and required diuretics.

##### Investigations

**Figure 6:**
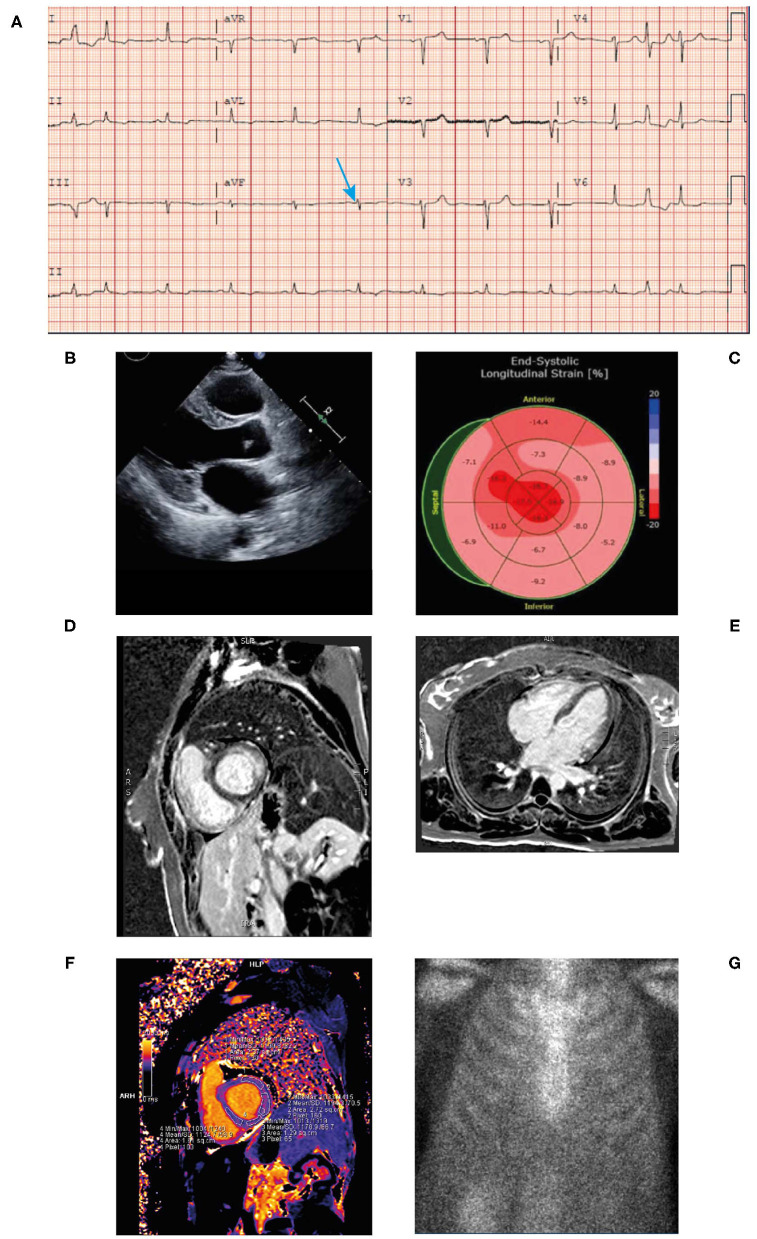
ECG **(A)** showing low voltage particularly in the limb leads (blue arrow), atrial premature contraction, sinus rhythm, and poor R-wave progression across the anterior precordial leads. Low voltage discordant with marked LVH. Echocardiogram **(B)** demonstrating concentric LVH, EF of 50% and left atrial enlargement. End-systolic longitudinal strain **(C)** showing markedly reduced global longitudinal strain pattern −11.8 with apical sparing. Cardiac MRI (+gadolinium) **(D,E)** revealing patchy late gadolinium enhancement of mid myocardial lateral wall base, inferior interventricular groove from base to mid ventricle and epicardial enhancement of the lateral wall, septal epicardial enhancement. Epicardial enhancement of entire RV free wall from base to apex. Cardiac MRI T1 mapping **(F)** showing mildly increased T1 value of left ventricle. Bone scintigraphy (^99m^Tc-PYP) planar 3-h scan **(G)** had Grade 0 uptake—not suggestive of ATTR. ATTR, transthyretin amyloidosis; ECG, electrocardiogram; EF, ejection fraction; LVH, left ventricular hypertrophy; MRI; magnetic resonance imaging; RV, right ventricular; TTR, transthyretin.

- Laboratory work-up revealed markedly **elevated troponin (0.074** **μg/L) and NT proBNP levels (1,556 ng/L)**; however, the patient did not exhibit signs of heart failure. Microcytic hypochromic anemia with hemoglobin 92 g/L and a hematocrit of 29%. Serum free kappa light chain titer was 7.93 mg/L; free lambda light chain titer was 1,217 mg/L; kappa-lambda ratio was 0.01. Serum immunofixation showed two separate bands of free lambda light chain specificity and urine immunofixation demonstrated free lambda light chain. All three measures of light chain were highly abnormal.
- Blood pressure was 88–92/50–54 mmHg.
- ECG revealed sinus tachycardia and marked diffuse **low voltage discordant with marked LVH on echocardiogram** (Figure 6A).
- **Bone marrow biopsy confirmed 25% monoclonal plasma cells with lambda light chain immunoglobulins**. Bone marrow biopsy was Congo red negative with no evidence of amyloid.
- Skin biopsy showed positive Congo red stain consistent with amyloidosis cutis and fat pad biopsy also confirmed the presence of amyloidosis.
- **An echocardiogram revealed mild concentric LVH** with a normal EF of 54% (Figure 6B). The left ventricular (LV) wall showed increased echogenicity.
- **Strain echocardiography revealed a decreased global longitudinal strain pattern (GLS-11.8) with relative apical sparing** that was highly suggestive of cardiac amyloidosis (Figure 6C).
- Cardiac MRI revealed patchy late gadolinium enhancement of fusiform mid-myocardial lateral wall base, enhancement of the inferior interventricular groove from base to mid ventricle, and epicardial enhancement of the lateral wall, epicardial RV free wall, and septal epicardial enhancement consistent with myocarditis. Mildly increased T1 value and extracellular volume (ECV) of the LV suggestive of mild myocardial edema (Figures 6D–F). From these imaging results, differential diagnoses include infiltrative disease and myocarditis. However, given the patient had LVH, diagnosis of infiltrative cardiomyopathy was highly suggestive.
- ° Increased ECV on T1 mapping is highly suspicious of AL with cardiac involvement in the setting of LVH and elevated light chains; the myocarditis was a misdiagnosis.
- ° AL protein deposition is highly toxic to the myocardium particularly at high titers and likely caused appearance of increased edema.
- Bone scintigraphy (^99m^Tc-PYP) had a Grade 0 uptake—-not suggestive of TTR amyloidosis (Figure 6G). Of note, in patients with AL amyloidosis the PYP scan is typically negative. The patient's presentation along with this finding should prompt an aggressive work up and bone marrow biopsy.

##### Diagnosis

AL systemic amyloidosis secondary to light chain myeloma with cardiac involvement.

##### Clinical Clues and Potential Pitfalls

This case highlights the need to integrate the clinical and potentially conflicting diagnostic modalities to establish the correct diagnosis. A typical clinical clue to the diagnosis of AL amyloidosis is the discordance between the negative PYP scan and the echocardiogram showing biventricular hypertrophy and GLS showing apical sparing pattern. It should be emphasized that in patients with AL amyloidosis the PYP scan is typically negative or low-grade uptake and clinicians should also be aware that the cardiac MRI may be misinterpreted as myocarditis. The typical physical findings in conjunction with abnormal light chains in a patient with progressive heart failure and markedly elevated BNP is highly suggestive of AL amyloidosis.

##### Outcome

Echocardiogram after 70 days revealed a drop in EF to 36%, increased myocardial echogenicity, GLS of−10.7, grade III left ventricular diastolic dysfunction and biatrial and biventricular enlargement. After the third cycle of chemotherapy, the patient was admitted to the hospital with worsening symptoms of heart failure and tested COVID-19 positive. Echocardiogram revealed an EF of 33% with a GLS of −6. NT proBNP had increased to >5,000 ng/L. A week later the patient was readmitted with worsening heart failure and hypokalemia and shortly after discharging herself against medical advice experienced a sudden cardiac arrest and died. The very rapid deterioration and fatal outcome in this young patient demonstrates that AL amyloidosis with cardiac involvement is a hematologic emergency.

#### Case 3: ATTR-CM Wild-Type (A Is Negative and B Is Positive)

##### Presentation

**Figure 7:**
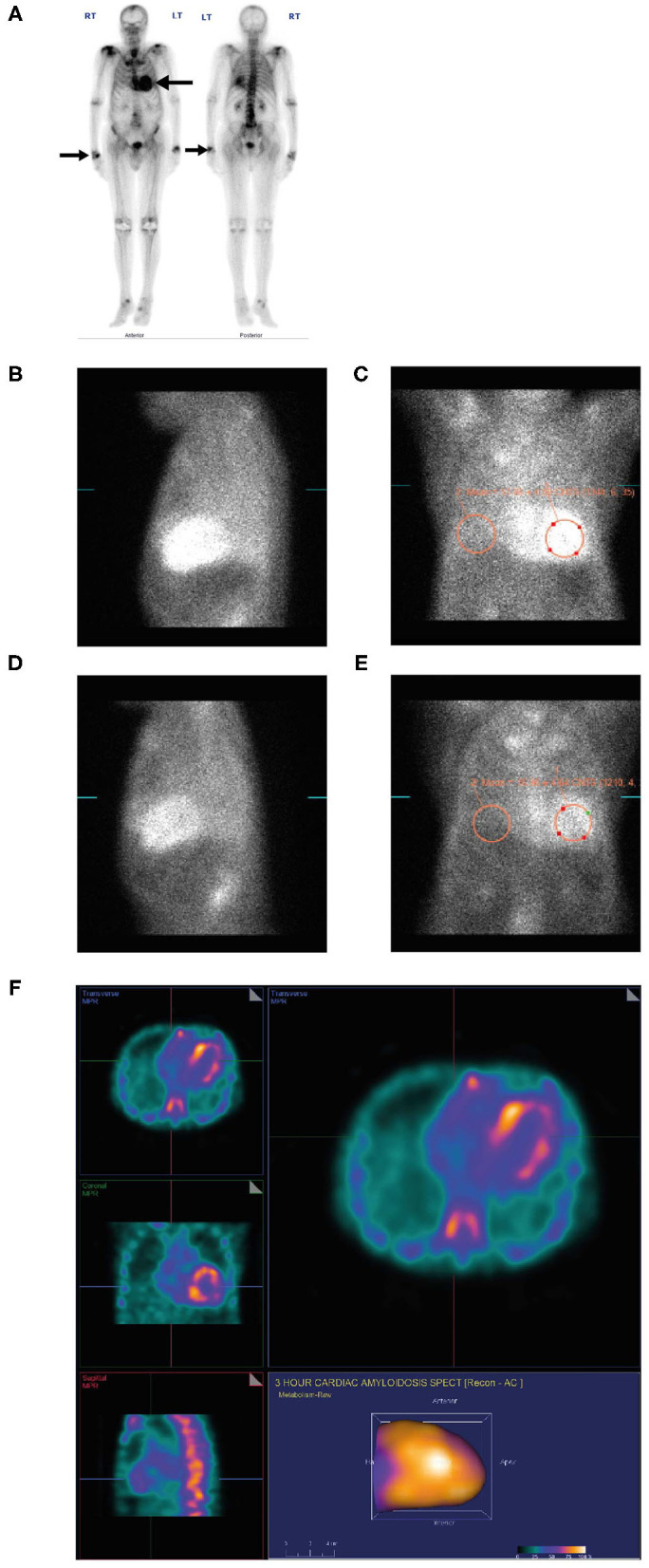
PYP scan **(A)** for metastatic prostate cancer showing an incidental finding of intense myocardial uptake of bone tracer (Grade 3, black arrow), greater than the ribs and spine and intense uptake in both wrists (black arrow) consistent with diagnosis of carpal tunnel syndrome. Lateral planar **(B)** and anterior planar **(C)** scans demonstrate intense cardiac PYP uptake at 1 h with H/CL ratio 1.67. At 3 h, lateral planar **(D)** and anterior planar **(E)** scans demonstrate intense cardiac PYP uptake with H/CL ratio 1.77. SPECT scan **(F)** demonstrates intense myocardial uptake in the RV and LV free walls with no blood pool in the left ventricular cavity. H/CL, heart to contralateral lung ratio; LV, left ventricular; PYP, pyrophosphate; RV, right ventricular; SPECT, single-photon emission computed tomography.

An **88-year-old man** with a long-standing history of walking difficulties, **spinal stenosis** and **lower limb edema** was referred to the cardiology department for pre-operative assessment for prostate cancer surgery due to his history of heart failure, **paroxysmal atrial fibrillation** and an abnormal ECG. He underwent an extensive work-up for ischemic cardiomyopathy, including coronary angiography. Over the following 18 months, serial echocardiograms were conducted during which his EF dropped progressively from 58 to 25% culminating in an admission for new onset of atrial fibrillation and decompensated heart failure. Due to symptomatic orthostatic hypotension, the patient was receiving sacubitril-valsartan 50 mg twice daily and spironolactone 12.5 mg once daily and was **intolerant of guideline-directed medications for heart failure**. He was seen by the heart failure team and ATTR was suspected. Recognizing the patient had a whole-body bone scan (ordered two years previously by his urologist for staging of metastatic prostate cancer) this was retrospectively reviewed revealing intense cardiac uptake of PYP bone tracer, greater than the ribs and spine (Grade 3) (Figure 7A) confirming the diagnosis.

##### Investigations

**Figure 8:**
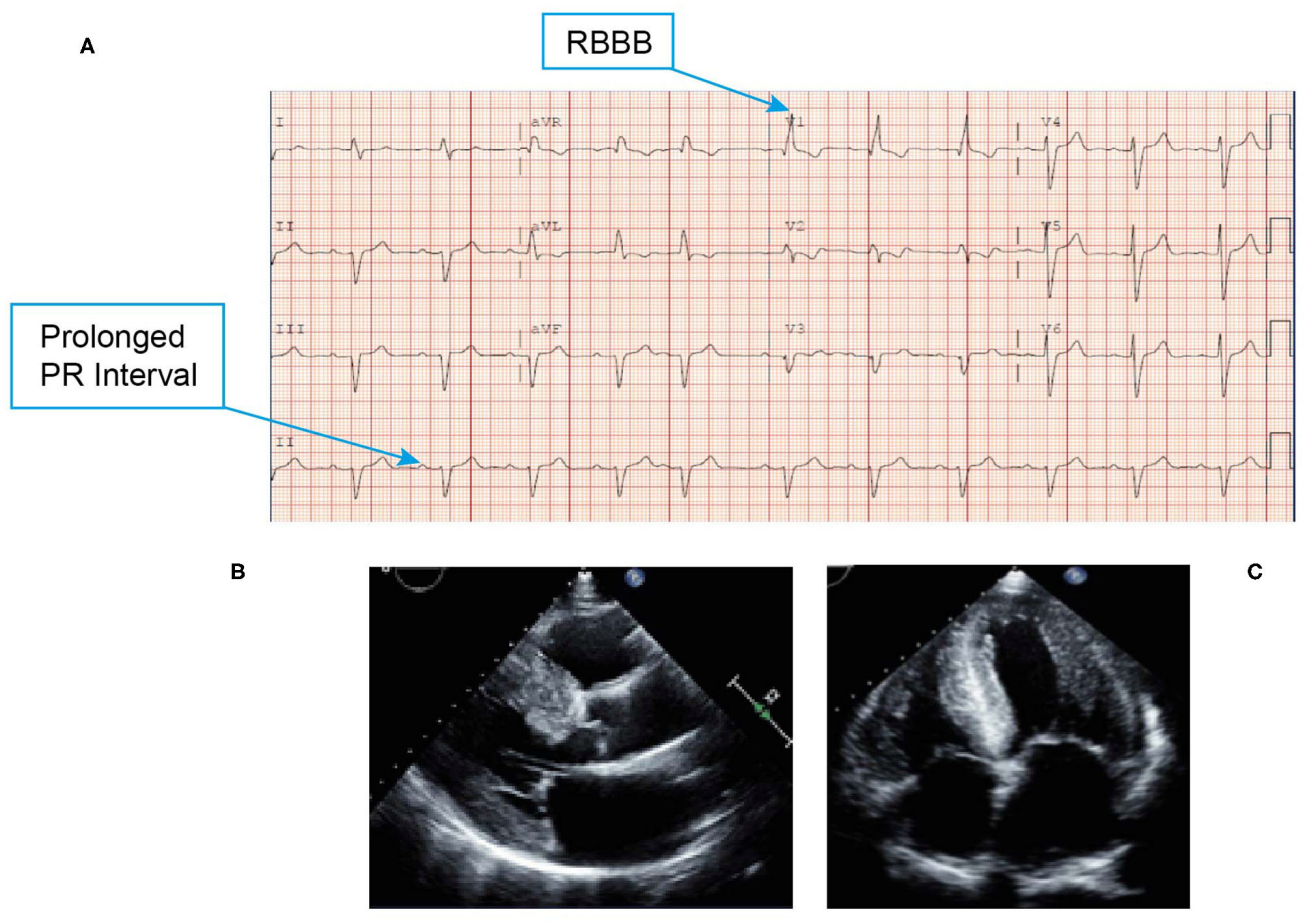
ECG **(A)** showing low voltage QRS, first-degree atrioventricular block and conduction system abnormality with PR interval prolongation with RBBB. Echocardiogram **(B,C)** revealing marked thickening of the left ventricle, right ventricle, and biatrial enlargement, and a classic speckled appearance. ECG, electrocardiogram; QRS, Q wave, R wave, and S wave; RBBB, right bundle branch block.

**Figure 9:**
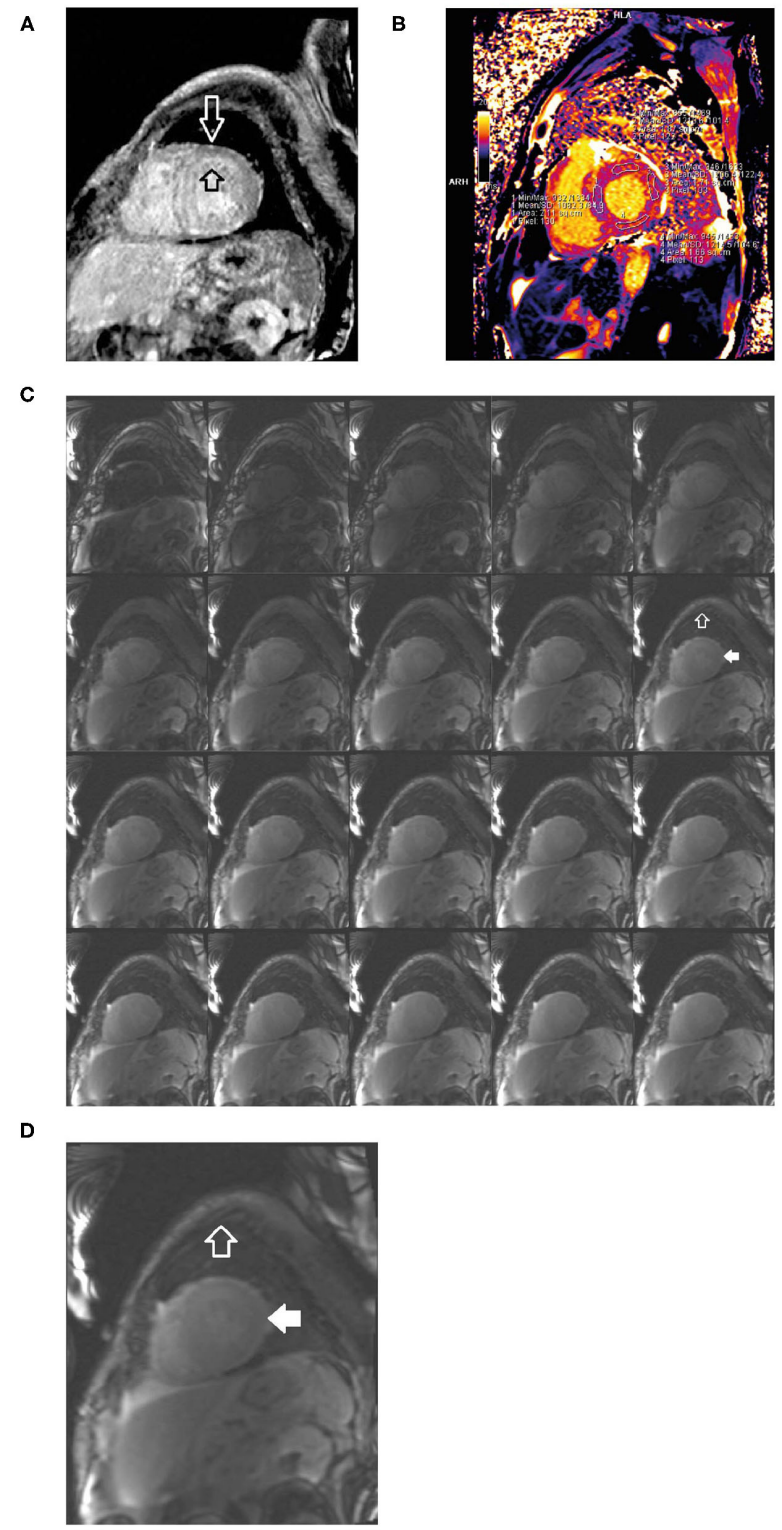
Short axis delayed enhancement MRI **(A)** demonstrates diffuse late enhancement of the circumferential hypertrophic left ventricle in a characteristic decreasing gradient from endocardium to epicardium (open black and white arrows). Cardiac MRI T1 mapping **(B)** shows increased T1 signal and ECV consistent with infiltration. Short axis composite layout of inversion time scout imaging **(C)** of the left ventricle, enlarged image **(D)**. In comparison to skeletal muscle (open white arrow), the myocardium is difficult to null (solid white arrow) because of the lack of normal (unenhanced) myocardium. The accumulation of the amyloid protein into which the gadolinium contrast equilibrates from the blood pool. ECV, extracellular volume; MRI, magnetic resonance imaging.

- Electromyography (EMG) was ordered by a neurologist and revealed **peripheral neuropathy**, symmetrical axonal sensory motor polyneuropathy with secondary demyelinating changes and **carpal tunnel syndrome**.
- An ECG revealed **low voltage QRS**, first-degree atrioventricular block (despite a **very thick left ventricle**) and conduction system abnormality with PR interval prolongation (290 ms) with **right bundle branch block (RBBB)** (Figure 8A). Discordance of LV voltage is seen during both atrial fibrillation and sinus rhythm.
- Coronary angiography revealed 30% distal left main stenosis and no significant epicardial coronary artery disease.
- An echocardiogram revealed a left ventricular EF of 40%, restrictive filling, and **reduced global strain with apical sparing and severe LVH** (Figures 8B,C).
- Laboratory work-up: Blood urea nitrogen 6.2 mmol/L, hemoglobin 119 g/L, creatinine 86 μmol/L, Ca 2.2 mmol/L, **troponin T 0.217** **μg/L, and NT proBNP 24,288 ng/L**. Serum and urine immunofixation electrophoresis were unremarkable. His kappa level was 51.06 mg/L, lambda level was 29.89 mg/dL, and kappa/lambda ratio was 1.71 (up to 2.5 is considered normal in patients with renal dysfunction [the patient had an eGFR of 59 mL/min/1.73m^2^] in the setting of normal serum and urine immunofixation electrophoresis) (31).
- An abdominal fat pad biopsy was negative for Congo red staining.
- A dedicated PYP scan revealed intense cardiac PYP uptake at 1 h (Figures 7B,C) and 3 h (Figures 7D,E) strongly suggestive of ATTR-CM with a markedly elevated H/CL ratio of 1.67 at 1 h and 1.77 at 3 h. A SPECT scan at 1 h revealed no blood pool (Figure 7F).
- Cardiac MRI revealed difficult nulling to the myocardium post contrast administration, diffuse delayed enhancement, LVH, and reduced left ventricular systolic function, there are elevated T1 and ECV values (Figure 9).

##### Diagnosis

Initially the patient's symptoms were attributed to possible ischemia and heart failure. Subsequent testing confirmed ATTR-CM.

##### Clinical Clues and Potential Pitfalls

This case highlights that clinical findings, especially neurological diagnoses and bone scan imaging data, in an elderly patient with progressive heart failure symptoms, progressive LV dysfunction with a thick ventricle, conduction system abnormality and atrial fibrillation should be integrated to reach a diagnosis. The presence of neurological symptoms prior to cardiac symptoms and the patient's elderly age is consistent with ATTRwt (26, 43). For this patient, the late stage at diagnosis caused the infiltrative process to progress with concomitant marked reduction in his ejection fraction. The imaging findings demonstrated diffuse late enhancement and failure to null. Alongside global left ventricular dysfunction, these classic advanced stage features of TTR amyloidosis may not be seen in earlier presentation of the disease. Ideally the patient should be diagnosed prior to this advanced stage, especially as the initial whole-body bone scan and echocardiograms contained clues for ATTR-CM.

#### Case 4: ATTR-CM Hereditary (Mutant) (A Is Negative and B Is Positive)

##### Presentation

A 55-year-old Irish American **man** previously living in Boston was evaluated for palpitations, **exertional dyspnea**, dizziness, **vertigo, fatigue** (worsening over 2 years), constipation, **generalized weakness** and a 2–3-month history of **weight loss**. He had severe **polyneuropathy** and bilateral weakness and had experienced episodes of **syncope**. He also had a history of paresthesia, **orthostatic hypotension, numbness** (starting with his left leg, spreading upwards), difficulty walking, orthostatic loss of consciousness, hoarseness of voice (starting 11 years prior), blurred vision, diarrhea, difficulty swallowing, **multiple upper GI investigations** and colonoscopies, muscular atrophy, sensory loss, weight loss (75 pounds over 2 years), and a 2-year history of erectile dysfunction. He had previously undergone **several orthopedic surgeries** (cervical spine, shoulder, spine). He had a strong family history of cancer and had a sibling with coronary artery disease.

##### Investigations

**Figure 10:**
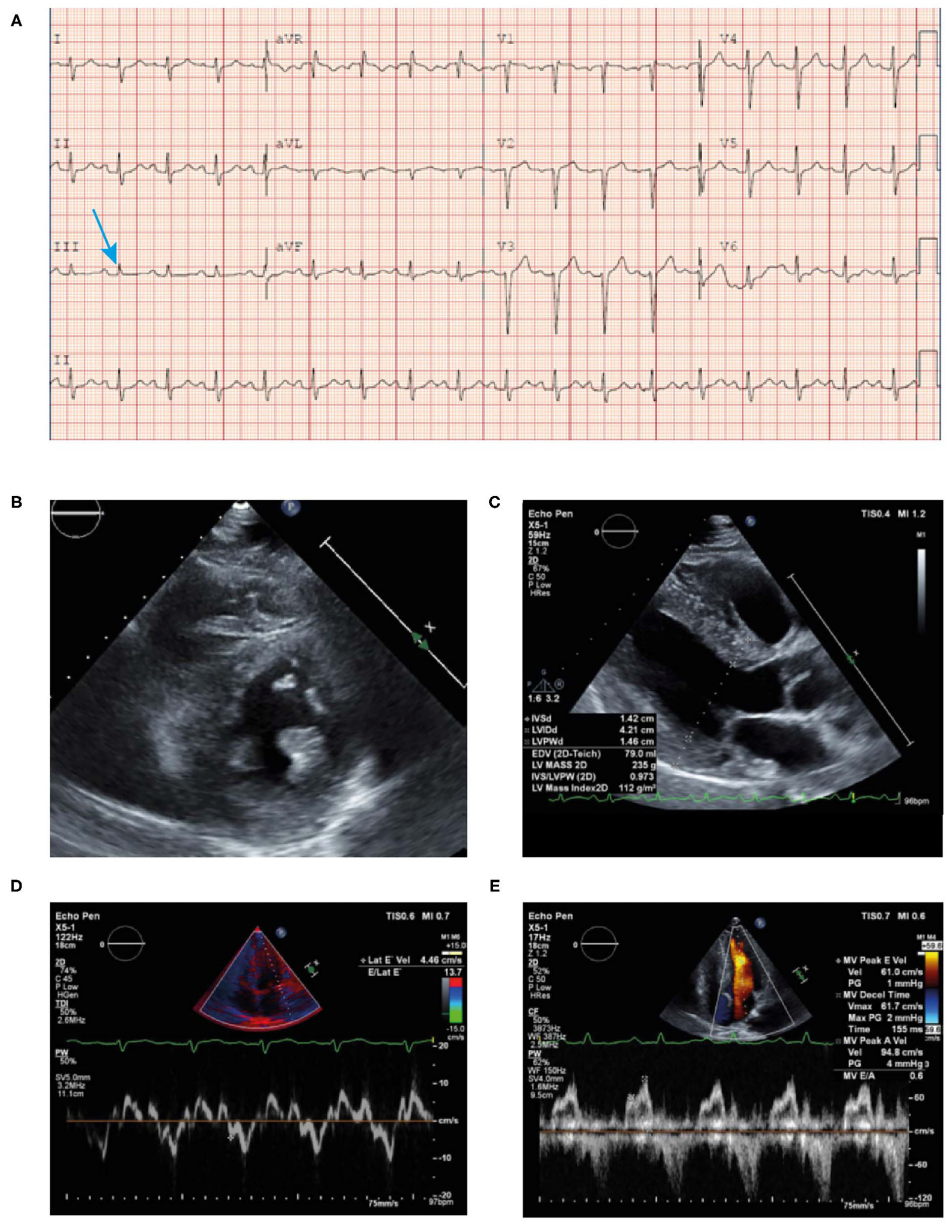
ECG **(A)** showing poor R wave progression in the anterior leads and prolonged PR interval consistent with first-degree AV block (arrow). Echocardiogram **(B–E)** findings showing unexplained concentric LVH (1.4 cm) with reduced lateral E velocity (4.46 cm/s) and mitral inflow consistent with diastolic dysfunction in the context of the ECG showing no evidence of LVH (discordance) in the absence of hypertension. ECG, electrocardiogram; LVH, left ventricular hypertrophy.

**Figure 11:**
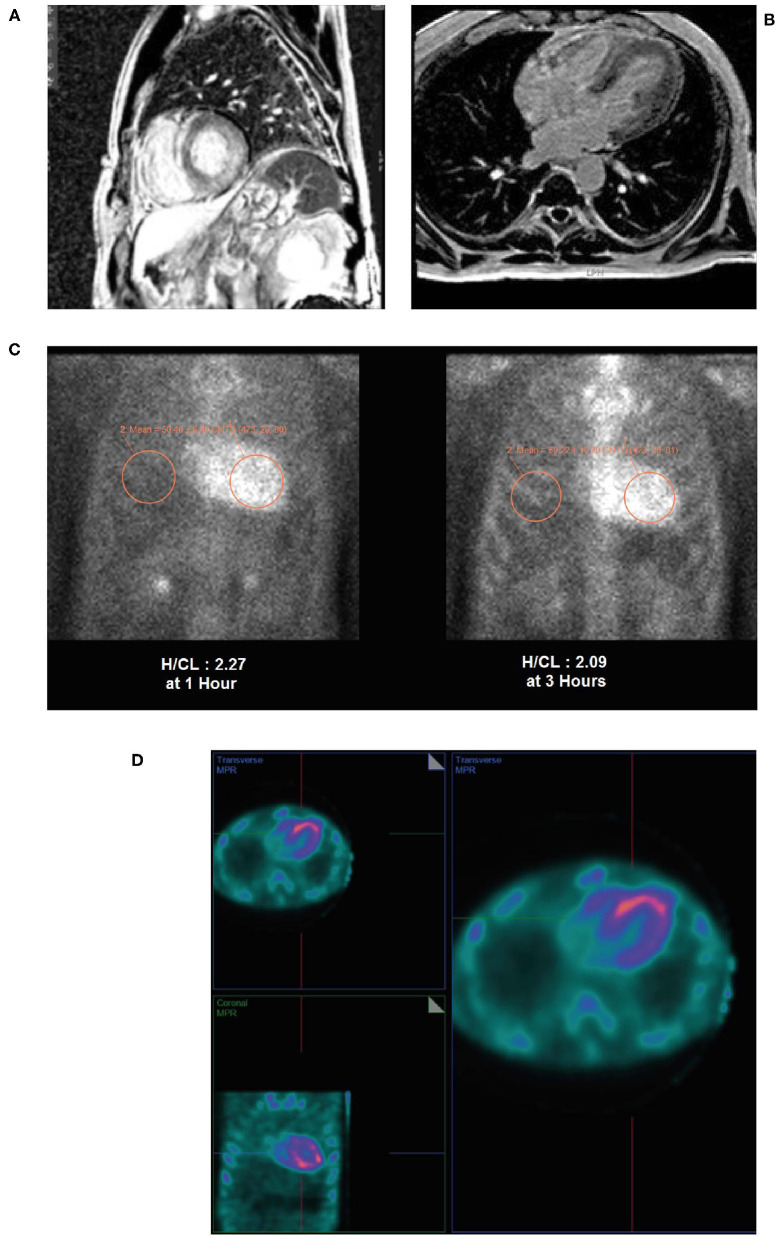
Cardiac MRI **(A,B)** showing patchy contrast enhancement pattern of the myocardium involving the left ventricular lateral wall base, and mid myocardium of the base to mid ventricle of the LV septum, and mid myocardium of the RV free wall from base to mid ventricle associated with hypertrophy. A planar PYP scan **(C)** revealed intense cardiac uptake at 1 h and 3 h (H/CL ratio 2.27 and 2.09, respectively), with SPECT scan **(D)** at 1 h demonstrating tracer uptake in myocardium and no residual LV blood pool activity. H/CL, heart to contralateral lung ratio; LV, left ventricular; MRI, magnetic resonance imaging; PYP, pyrophosphate; RV, right ventricular; SPECT, single-photon emission computed tomography.

- An ECG revealed borderline **prolongation of the PR interval** (Figure 10A).
- An echocardiogram revealed a normal size left ventricle with moderate concentric LVH (Figures 10B–E). The hypertrophy was disproportional to age and there was discordance between the left ventricle and absence of evidence of LVH on the ECG. Left ventricular systolic function was normal. The right ventricle was normal in size and systolic function. There was evidence of myocardial or infiltrative disease.
- Cardiac MRI revealed a patchy contrast enhancement pattern of the myocardium involving the left ventricular lateral wall base, and mid myocardium of the base to mid ventricle of the LV septum, and mid myocardium of the RV free wall from base to mid ventricle associated with hypertrophy; this pattern is highly concerning for **advanced cardiac sarcoid** (Figures 11A,B).
- Laboratory work-up: Blood urea nitrogen (BUN) 5.0 mmol/L, hemoglobin 158.0 g/L, creatinine 99.0 μmol/L, Thyroid function test (TSH) 1.5 IU/L, troponin T 0.020 μg/L, NT proBNP 287.7 ng/L. Serum kappa 10.50 mg/L, lambda 12.64 mg/L, Serum kappa/lambda ratio 0.83. Free serum light chain (kappa/lambda ratio) was in the normal range showing no evidence of AL amyloidosis. Serum and urine immunofixation were both negative.
- A subsequent PYP scan showed radiotracer uptake strongly suggestive of TTR cardiac amyloid (Figure 11C).
- A SPECT scan at 1 h revealed markedly intense uptake in the left ventricle myocardium with no blood pool at 1 h (Figure 11D).
- Genetic testing was subsequently undertaken at Columbia University, USA, showing a *TTR* Thr60Ala mutation that is typically seen in patients with Irish ancestry and almost always affects the heart with polyneuropathy.

##### Diagnosis

The patient was diagnosed with hereditary ATTR-CM.

##### Clinical Clues and Potential Pitfalls

Amyloidosis can have different patterns of uptake on cardiac MRI that may not be typical and can be confused with other infiltrative cardiomyopathies. The differential diagnosis should always pursue amyloidosis if there are cardiac, GI, and neurological symptoms in a young patient. Sarcoidosis is very unlikely due to absence of lung, lymph nodes or skin involvement. In contrast, PYP scan is highly diagnostic.

## Discussion

Cardiac amyloidosis carries a significant disease burden for patients and society particularly as this life-threatening condition is often mis- or under-diagnosed. Recent advances in the understanding of cardiac amyloidosis and improved diagnostic tools mean that in the majority of cases it is now possible to diagnose this condition early and allows treatment for ATTR-CM to be initiated without undue delay.

This publication assembles, in an easily accessible format, all elements that are needed for the clinical suspicion of cardiac amyloidosis. Disease awareness itself is a major determinant for an early diagnosis. The constellation of clinical signs and symptoms are clearly summarized in the “red flags” mnemonic and the diagnostic algorithm simplifies the complicated diagnostic process. The illustrative cases demonstrate how to search for the “red flag” clues in real-world patient cases and how to use the diagnostic algorithm to allow clinicians to arrive at a diagnosis of ATTR-CM. The clinical clues and potential pitfalls of diagnosing this disease have been highlighted in each case to provide a further learning tool.

The use of non-invasive diagnostic tools for ATTR-CM allow clinicians to avoid endomyocardial biopsy which is an invasive procedure that carries a risk for serious complications and requires technical expertise (13, 34). There are instances where endomyocardial biopsy is required to establish the diagnosis. Firstly, when AL amyloidosis cannot be excluded; secondly, in situations with a negative or equivocal ^99m^Tc-PYP scan to confirm ATTR-CM despite a high clinical suspicion, and lastly when ^99m^Tc-PYP scanning is unavailable (1).

Given the availability of effective therapy for ATTR-CM and AL amyloidosis, any delay in the diagnosis and subsequent treatment has an impact on mortality and morbidity and should be avoided. Where possible, family members of patients with hereditary ATTR-CM should be presented with the opportunity to undergo genetic testing for early identification of ATTR-CM to ensure early diagnosis and any subsequent treatment.

It is imperative that clinicians are vigilant when it comes to identifying patients to screen for ATTR-CM and that they familiarize themselves with the many clinical symptoms or “red flags” associated with this disease.

## Data Availability Statement

The original contributions presented in the study are included in the article/supplementary material, further inquiries can be directed to the corresponding author/s.

## Ethics Statement

Written informed consent was obtained from the individual(s) for the publication of any potentially identifiable images or data included in this article.

## Author Contributions

All authors made a significant contribution to the work reported, whether that is in the conception, acquisition of data, analysis and interpretation, in all these areas, took part in drafting, revising, critically reviewing the article, gave final approval of the version to be published, have agreed on the journal to which the article has been submitted, and agree to be accountable for all aspects of the work.

## Conflict of Interest

HS, HA, and FA reports personal fees from Pfizer Gulf FZ LLC, outside the submitted work. IR was a full-time employee of Pfizer Gulf LLC. This work was supported by Pfizer Gulf FZ LLC. Pfizer provided funding for the editorial assistance in the development of the manuscript. Neither honoraria nor payments were made for authorship. The remaining authors declare that the research was conducted in the absence of any commercial or financial relationships that could be construed as a potential conflict of interest.
